# Somatic mutation and gain of copy number of *PIK3CA *in human breast cancer

**DOI:** 10.1186/bcr1262

**Published:** 2005-05-31

**Authors:** Guojun Wu, Mingzhao Xing, Elizabeth Mambo, Xin Huang, Junwei Liu, Zhongmin Guo, Aditi Chatterjee, David Goldenberg, Susanne M Gollin, Saraswati Sukumar, Barry Trink, David Sidransky

**Affiliations:** 1 Department of Otolaryngology-Head and Neck Surgery, Head and Neck Cancer Research Division, Johns Hopkins University School of Medicine, Baltimore, MD, USA; 2 Division of Endocrinology and Metabolism, Department of Medicine, Johns Hopkins University School of Medicine, Baltimore, MD, USA; 3 Department of Human Genetics, University of Pittsburgh Graduate School of Public Health, Pittsburgh, PA, USA; 4 Breast cancer program, The Sidney Kimmel Comprehensive Cancer Center at Johns Hopkins, Baltimore, MD, USA

## Abstract

**Introduction:**

Phosphatidylinositol 3-kinases (PI3Ks) are a group of lipid kinases that regulate signaling pathways involved in cell proliferation, adhesion, survival, and motility. Even though *PIK3CA *amplification and somatic mutation have been reported previously in various kinds of human cancers, the genetic change in *PIK3CA *in human breast cancer has not been clearly identified.

**Methods:**

Fifteen breast cancer cell lines and 92 primary breast tumors (33 with matched normal tissue) were used to check somatic mutation and gene copy number of *PIK3CA*. For the somatic mutation study, we specifically checked exons 1, 9, and 20, which have been reported to be hot spots in colon cancer. For the analysis of the gene copy number, we used quantitative real-time PCR and fluorescence *in situ *hybridization. We also treated several breast cancer cells with the *PIK3CA *inhibitor LY294002 and compared the apoptosis status in cells with and without *PIK3CA *mutation.

**Results:**

We identified a 20.6% (19 of 92) and 33.3% (5 of 15) *PIK3CA *somatic mutation frequency in primary breast tumors and cell lines, respectively. We also found that 8.7% (8 of 92) of the tumors harbored a gain of *PIK3CA *gene copy number. Only four cases in this study contained both an increase in the gene copy number and a somatic mutation. In addition, mutation of *PIK3CA *correlated with the status of Akt phosphorylation in some breast cancer cells and inhibition of *PIK3CA*-induced increased apoptosis in breast cancer cells with *PIK3CA *mutation.

**Conclusion:**

Somatic mutation rather than a gain of gene copy number of *PIK3CA *is the frequent genetic alteration that contributes to human breast cancer progression. The frequent and clustered mutations within *PIK3CA *make it an attractive molecular marker for early detection and a promising therapeutic target in breast cancer.

## Introduction

Phosphatidylinositol 3-kinases (PI3Ks) are a group of lipid kinases composed of 85-kDa and 110-kDa subunits. The 85-kDa subunit lacks PI3K activity and acts as adaptor, coupling the 110-kDa subunit (P110) to activated protein tyrosine kinases and generating second messengers by phosphorylating membrane inositol lipids at the D3 position. The resulting phosphatidylinositol derivatives then permit activation of downstream effectors that are involved in cell proliferation, survival, metabolism, cytoskeletal reorganization, and membrane trafficking [1,2].

*PIK3CA*, the gene encoding the 110-kDa subunit of PI3K, was mapped to 3q26, an area amplified in various human cancers including ovarian, head and neck, breast, urinary tract, and cervical cancers [3-5]. *PIK3CA *was specifically found to be amplified and overexpressed in ovarian and cervical cancer [6-9]. The increased copy number of the *PIK3CA *gene is associated with increased *PIK3CA *transcription, P110-alpha protein expression, and PI3K activity in ovarian cancer [9]. Treatment with a PI3K inhibitor decreased proliferation and increased apoptosis, suggesting that *PIK3CA *has an important role in ovarian cancer. More recently, *PIK3CA *mutations were identified in different human cancers. In that report, *PIK3CA *was mutated in 32%, 27%, 25%, and 4% of colon, brain, gastric, and lung cancers, respectively. Only 12 cases of breast cancer were examined, of which one was found to harbor a mutation in *PIK3CA *[10].

In an effort to identify the genetic alterations of the *PIK3CA *gene in breast cancer, we determined the mutation frequency and the change in the gene copy number of *PIK3CA *in a set of primary breast tumors and breast cancer cell lines. We found a high frequency of these somatic alterations of *PIK3CA *gene in a large number of primary breast cancers. In addition, mutation of the *PIK3CA *gene correlated with the activation of Akt. Inhibition of *PIK3CA *induced significant apoptosis in cells with *PIK3CA *mutation.

## Materials and methods

### Breast cancer cell line and tumors

Of the breast cancer cell lines examined, MCF12A, Hs.578t, and MDA436 were kindly provided by Dr Nancy Davidson at Johns Hopkins University, and MDA-MB157, MDA-MB468, BT474, T47D, and UACC893 were kindly provided by Dr Fergus J Couch at Mayo Clinic. The other cell lines were obtained from the American Type Culture Collection. A total of 92 cases of breast tumor, including 33 paired primary invasive breast carcinomas and adjacent normal tissues (frozen tissue), were obtained from the Surgical Pathology archives of the Johns Hopkins Hospital, Baltimore, MD, USA, in accordance with the Institutional Review Board protocol and DNA was isolated using a standard phenol–chloroform protocol. Prof Saraswati Sukumar at the Sidney Kimmel Comprehensive Cancer Center at Johns Hopkins University provided isolated DNA. Each tumor used in this study was determined to contain greater than 70% tumor cells by H&E staining. Among these specimens, 3 were stage 1, 52 were stage 2, 22 were stage 3, and 4 were stage 4. Eleven were of uncharacteristic stage status. All of the tumors were high grade.

### PCR, sequencing, and mutational analysis

Cell line and tumor DNA were isolated as standard protocol. The primers we used for PCR and sequencing were as follows. For exon 1: forward, CTCCACGACCATCATCAGG, reverse, GATTACGAAGGTATTGGTTTAGACAG, and sequencing primer, ACTTGATGCCCCCAAGAATC; for exon 9: forward, GATTGGTTCTTTCCTGTCTCTG, reverse, CCACAAATATCAATTTACAACCATTG, and sequencing primer, TTGCTTTTTCTGTAAATCATCTGTG; for exon 20: forward, TGGGGTAAAGGGAATCAAAAG, reverse, CCTATGCAATCGGTCTTTGC, and sequencing primer, TGACATTTGAGCAAAGACCTG. We used the same PCR conditions for all three exons. After incubation at 95°C for 5 min, two cycles of amplification were performed at the initial annealing temperature of 62°C, with a subsequent annealing temperature decrease of 2°C for every two cycles until 54°C. Twenty-five amplification cycles were then performed. After PCR reaction, samples were subjected to automated DNA sequencing using the ABI 377 Sequencer. The positive samples were confirmed by re-PCR and sequencing using the same primers and conditions.

### Western blotting

To evaluate Akt phosphorylation status, MDA231, MD361, MCF7, BT20, BT474, and T47D cells were grown in appropriate medium and cell lysates were collected in SDS lysis buffer (cell signaling). Lysates were cleared of insoluble material by microcentrifugation at 15,800 *g *for 15 min at 4°C, and protein concentrations were determined (protein assay kit; Bio-Rad, Hercules, CA, USA). Approximately 50 μg of total protein from each sample was denatured in loading buffer for 10 min, electrophoresed through 10% polyacrylamide gels, and electroblotted to a nylon transfer membrane (Schleicher & Schuell, Bioscience, Keene, NH USA). The membrane was incubated overnight with primary antibody Akt ser473 (antirabbit, cell signaling), Akt (anti rabbit, Cell signaling) or β-actin (antimouse antibody; Sigma, St Louis, MO, USA) at 4°C. Then the membrane was washed three times in Tris-buffered saline with 0.1% Tween 20 at room temperature and incubated for 1 hour at room temperature with horseradish-peroxidase-labeled secondary antibody (goat antirabbit IgG; or goat antimouse IgG; Sigma). Signal detection was by horseradish peroxidase chemiluminescent reaction (ECL; Amersham).

### Quantitative real-time PCR

For real-time PCR, specific primers and probes were designed using software from Applied Biosystems (Foster City, CA, USA) to amplify the *PIK3CA *and control β-actin (sequences are available on request). Using this combination and the protocol described by Mambo and colleagues [11], the samples were run in triplicate. Primers and probes to β-actin were run in parallel to standardize the input DNA (4 ng). Standard curves were developed using serial dilutions of DNA extracted from MCF12A. PCR amplifications were performed on an ABI 7900 TaqMan (Applied Biosystems) according to the manufacturer's protocol.

### Fluorescence *in situ *hybridization (FISH)

Bacterial artificial chromosome (BAC) clone RP11-466H15 for *PIK3CA *was obtained from Research Genetics (Invitrogen Corporation, Carlsbad, CA, USA). BAC DNA isolation was carried out using the standard laboratory protocol for phenol–chloroform extraction. The chromosome 3 α-satellite plasmid and BAC DNA were labeled directly in SpectrumOrange-dUTP^® ^and SpectrumGreen-dUTP^® ^(Vysis, Downers Grove, IL, USA), respectively, using the Vysis nick translation kit (Vysis) in accordance with the manufacturer's instructions. Slides were fixed using methanol:acetic acid (3:1), followed by pretreatment with RNase, and dual-color FISH was performed as described previously [12]. Slides were counterstained with 4',6-diamidino-2-phenylindole (DAPI; Sigma), mounted with antifade (Vysis), and stored at -20°C. At least 100 nuclei were evaluated for each sample. Analysis was carried out using an Olympus (New Hyde Park, NY, USA) BHS fluorescence microscope, and images were captured using a CytoVision Ultra (Applied Imaging, Santa Clara, CA, USA).

### Apoptosis detection

We assessed cellular apoptosis using an Annexin V-FITC (fluorescein isothiocyanate) apoptosis detection kit (BD Biosciences, San Jose, CA, USA). Cells were cultured in 100-mm dishes until 50% confluent, serum-starved overnight, and then treated with LY294002, 3 μM and 10 μM, for 72 hours. Both detached and adherent cells were then collected and labeled with Annexin V-FITC and Propidium Iodide. The apoptosis was evaluated using FACScan (Becton Dickinson ImmunoSystems, Mountain View, CA, USA) flow cytometer.

## Results

### *PIK3CA *is frequently mutated in breast cancer cell lines and primary tumors

**Figure 1:**
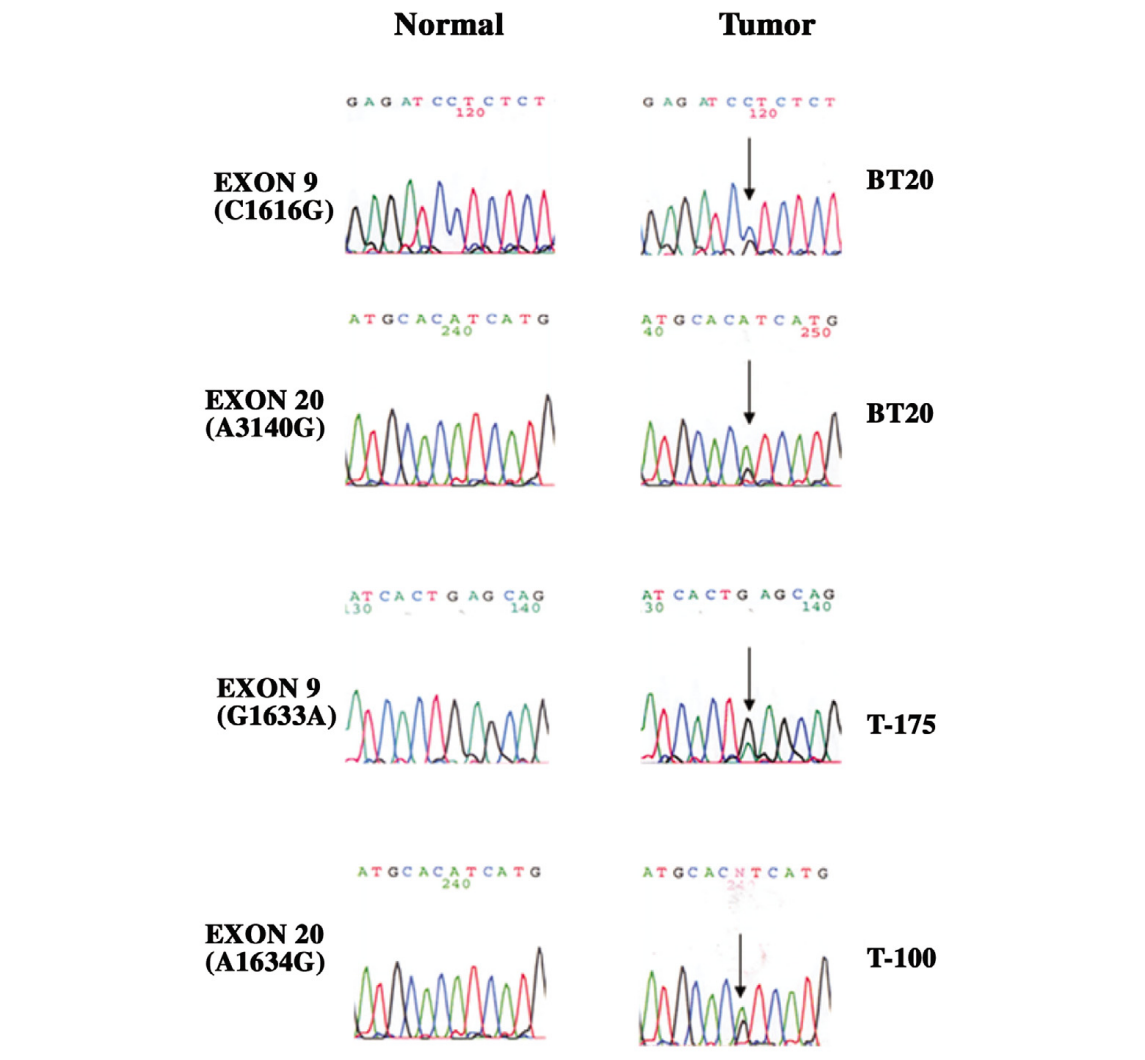
Detection of somatic mutation of *PIK3CA *in breast cancer. In each case, the left sequence chromatogram was obtained from normal control and the right sequence chromatogram was obtained from tumor. Arrows indicate the location of missense mutations. The nucleotide and amino acid alterations are indicated on the left.

A previous report suggested that more than 80% of the mutations of the *PIK3CA *gene occur in three small clusters, namely in the p85 (exon 1), helical (exon 9) and kinase (exon 20) domains [10]. Based on this information, we sequenced exon 1, 9, and 20 in 15 breast cancer cell lines, 92 primary tumors, and 33 normal tissues. A total of five mutations were identified only in the 15 breast cancer cell lines (33.3%). No mutations were detected in the normal epithelial cell line MCF12A. Three of the mutations were identified in exon 9 and two were found in exon 20. No mutation was identified in exon 1. The BT20 cell line contained two different mutations, C1616G in exon 9 and A3140G in exon 20 (Fig. 1), corresponding to P539R and H1047R amino acid change, respectively.

A total of 19 mutation cases (20.6%) were identified in 92 primary tumors. Six of the 19 mutations were identified in 33 tumor samples but not in their paired normal samples. This indicated that the identified mutations are somatic mutations. Thirteen of these 19 mutations were in exon 9, and 6 were in exon 20. No mutation was identified in exon 1 in any of the 92 tumors. As shown in Table 1, the E545K mutation in exon 9 and the H1047R mutation in exon 20 were the two most frequent mutations in both breast cancer cell lines and primary tumors.

### Gain of copy number of *PIK3CA *gene in primary breast cancer cell lines and tumors

**Figure 2:**
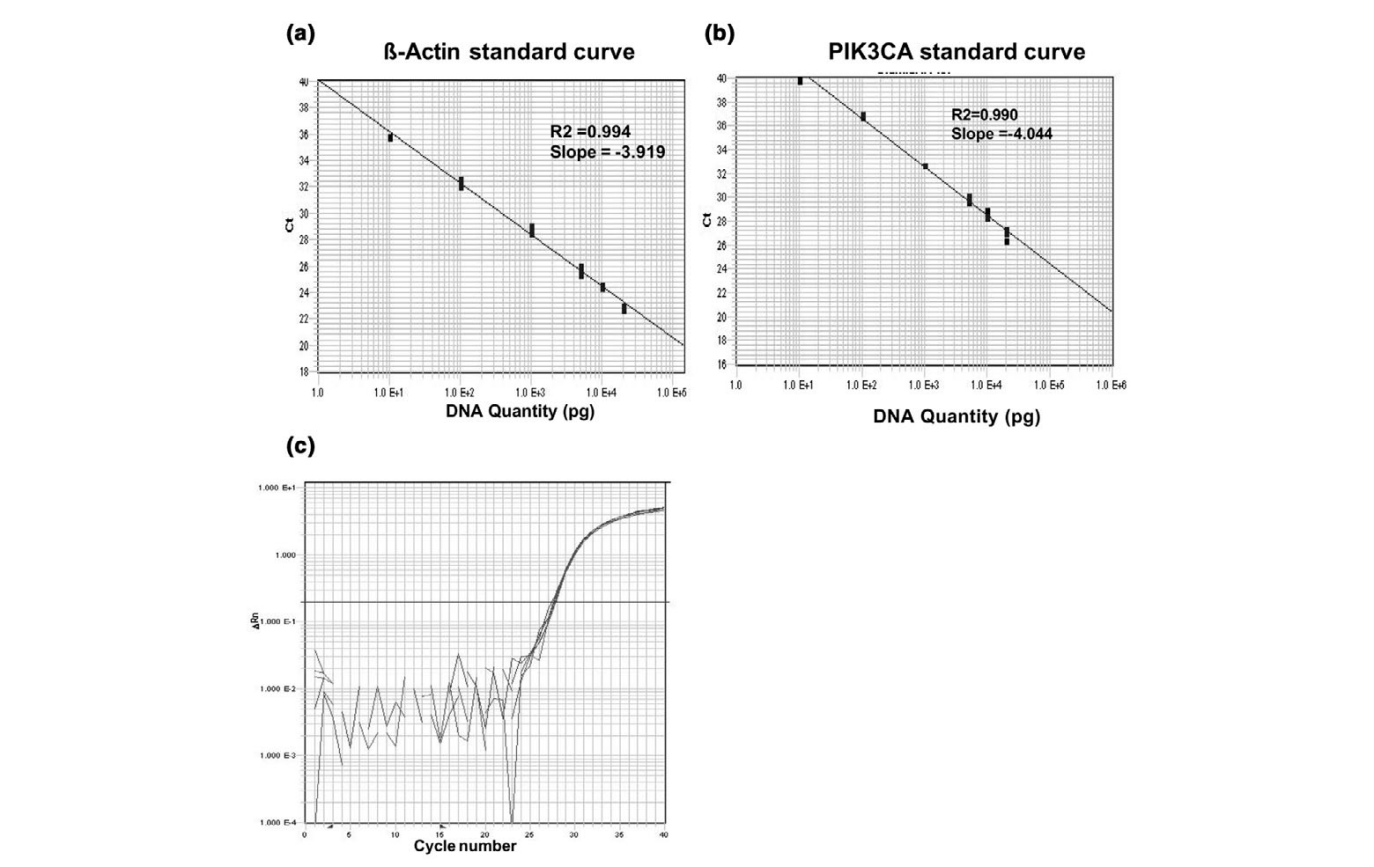
Typical real-time PCR curves generated for a cell line, a tumor, and normal tissue. Typical standard curves generated for **(a) **β-actin and **(b) ***PIK3CA *using serial diluted DNA from cell line MCF12A. **(c) **Representative real-time PCR curves for β-actin, generated using paired normal and tumor DNA from breast tissue of one individual. Each experiment was performed in triplicate and is shown by overlapping amplification curves. ΔRn = (Rn^+^) - (Rn^-^), where Rn^+ ^is the fluorescence emission intensity of reporter/emission intensity of quencher at any time point, and Rn^- ^is the initial emission intensity of reporter/emission intensity of quencher in the same reaction vessel before PCR amplification was initiated. Ct, cycle threshold.

**Figure 3:**
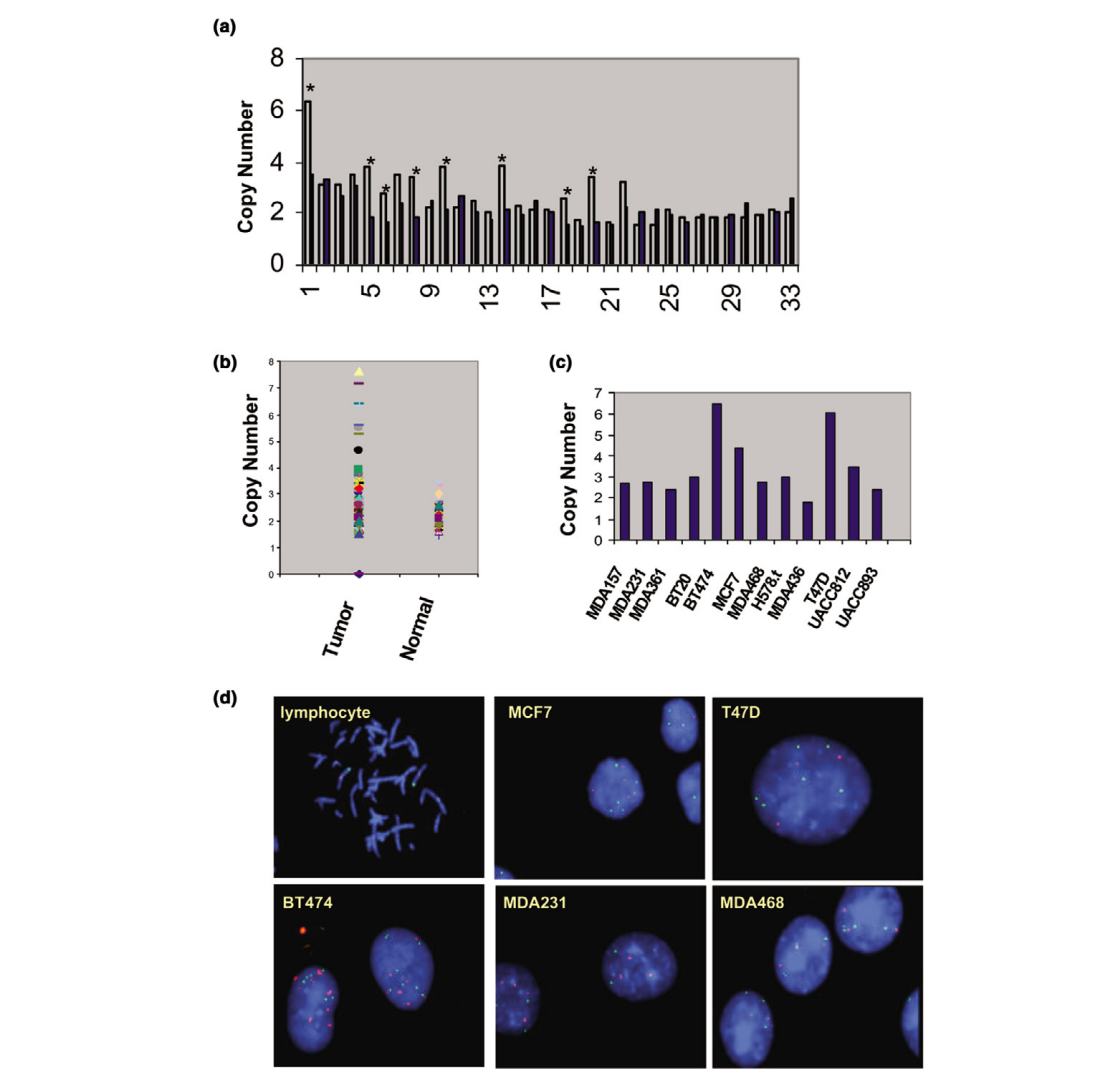
Gain of *PIK3CA *gene copy number in breast cancer. **(a)**The change in the gene copy number of *PIK3CA *in 33 paired breast tissue samples. *Cases with significant change in gene copy number. The case number is indicated below the panel. **(b) **The change in the gene copy number of *PIK3CA *in 92 breast tumors and 33 normal controls. **(c) **Eight samples show more than 4 copies of *PIK3CA*. **(d) **The change in the gene copy number of *PIK3CA *in breast cancer cells using real-time PCR. Representative images of fluorescence *in situ *hybridization (FISH) analysis in various breast cancer cell lines. Green signals represent bacterial artificial chromosome (BAC) 466H15 probe. Red signals represent chromosome 3 centromere probe.

To determine the *PIK3CA *gene copy number, we performed real-time quantitative PCR on 12 breast cancer cell lines, 92 primary tumors, and 33 normal controls. Standard curves for *PIK3CA *and β-actin amplification were generated using serially diluted MCF12A DNA, and showed linearity over the range used. Fig. 2a shows the standard curve for *PIK3CA *amplification with a slope of -4.044, while Fig. 2b shows the standard curve for β-actin amplification with a slope of -3.919. We did not observe any deletion of β-actin in the tumor samples. Most samples showed no difference in β-actin amplification between the paired tumor and a normal samples. A representative figure of β-actin amplification in a paired tumor and normal sample is shown in Fig. 2c. To evaluate the gene copy number in all samples, we set the cutoff line at 4 copies. Among the 33 cases with paired tissue, 8 (24.2%) showed a much higher gene copy number than normal controls (Fig. 3a). Only one case showed more than 4 copies. In a total of 92 cases of primary tumors, 8 (8.7%) had more than 4 copies, with the highest number being 7.8 copies (Fig. 3b). In addition, *PIK3CA *gene copy number was also determined in 12 breast cancer cell lines, and the MCF7, T47D, and BT474 cell lines had more than 4 copies (Fig. 3c). We also confirmed the gene copy number results of these 12 cell lines with FISH analysis. Representative FISH images are shown in Fig. 3d. Thus, our data of gene copy analysis indicates that gene amplification/gain of copy number of *PIK3CA *gene is not a frequent genetic alteration in breast cancer.

### Biological effect of *PIK3CA *mutations in breast cancer

**Figure 4:**
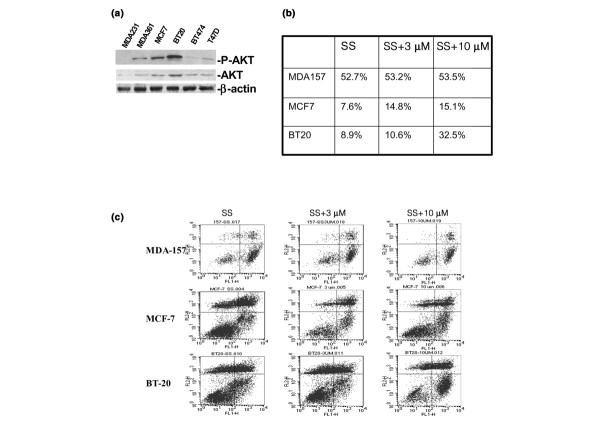
Biological effect of *PIK3CA *mutations in breast cancer. **(a) **Phosphorylation (P) of Akt in breast cancer cells. Western blotting showed stronger phosphorylation of Akt in BT20 and MCF7 cells than in MDA 231, MDA361, BT474, or T47D. β-Actin was used as a protein loading control. **(b) **Summary of the fraction of apoptotic cell in three cell lines with different treatments (3 μM and 10 μM LY294002). **[(c) **Apoptosis measurement in which cells were stained with Annexin V-FITC and for DNA content with Propidium Iodide and analyzed using flow cytometry. Apoptotic cells appear as a discrete population with elevated FITC fluorescence. FITC, fluorescein isothiocyanate; SS, serum starvation

To determine whether the mutation of *PIK3CA *correlated with the activation of *Akt *(a downstream gene of PIK3 that mediates carcinogenic events such as proliferation), we performed western blot analysis to check the phosphorylation of Akt in several breast cancer cell lines. As shown in Fig. 4a, Akt phosphorylation was strongest in BT20 cells (which harbor two *PIK3CA *mutations) and MCF7 cells (which harbor *PIK3CA *mutation and high *PIK3CA *gene copy numbers). We also observed weak phosphorylation of Akt in MDA361 (which has one mutation) and in BT474 and T47D (no observable mutation but with high *PIK3CA *gene copy numbers). We did not observe phosphorylation of Akt in MDA231 (Fig. 4a) or in MCF12A and MDA157 cells (data not shown) that had no observable mutations and no copy number gain of *PIK3CA*. These data indicate that *PIK3CA *mutations might increase kinase activity and in turn activate the PI3K/AKT pathway.

We further investigated the biological effects of *PIK3CA *mutation in breast cancer cell lines by treating breast cancer cells with or without *PIK3CA *mutation with the *PIK3CA *inhibitor LY294002. As shown in Table 1, MCF7 harbors one mutation at position E545K, and BT20 harbors two mutations, which are located at positions P539R and H1047R. These somatic mutations were recently shown to have oncogenic transforming activity [13]. As shown in Fig. 4b and 4c, the fractions of apoptotic cells at 72 hours after treatment with 3 μM and 10 μM LY294002 were increased in MCF7 and BT20 cells. In addition, 3 μM and 10 μM LY294002 did not induce further apoptosis in MDA157 cells (Fig. 4c) or MDA231 cells (data not shown), even though serum starvation alone can induce more than 50% apoptosis in MDA157 cells (Fig. 4c).

## Discussion

This study describes two innovations. First, we show a 20.6% mutation rate of the *PIK3CA *gene in breast cancer, indicating that *PIK3CA *mutation is a frequent genetic alteration in breast cancer. The 8% mutation rate of *PIK3CA *in breast cancer, reported in a previous study, was underestimated [10], probably because of the smaller number of cases examined. Another possibility might be the grade status of the tumors used, as all of the tumors in our study were of high grade. It will be useful and interesting in the future to explore whether *PIK3CA *mutation is correlated with tumor grade status.

Second, CGH (comparative genomic hybridization) studies have shown that 3q26 is an amplified chromosome region in various cancers, including breast cancer [4,5]. Unfortunately, it was not previously possible to identify the *PIK3CA *gene amplification pattern, because of the low resolution of the methods used. In our study, we used quantitative real-time PCR, a very sensitive and far more accurate technique [14,15], to specifically quantitate the genomic copy number of *PIK3CA *not only in primary breast tumors but also in paired tissues. Our data showed that gene amplification or gain of *PIK3CA *copy number is not a frequent genetic alteration event. This suggests that gene amplification is not the main molecular mechanism in activating the PIK3/AKT-driven tumorigenesis pathway in breast cancer.

Even though a complex and heterogeneous set of genetic alterations, including gene amplification/gain of copy number, deletion, and mutation, were reported to be involved in the etiology of breast cancer [16,17], our paper confirmed that gain of gene copy number and somatic mutation of one oncogene exist in parallel in breast cancer. Both amplification/gain of gene copy number and somatic mutation of *PIK3CA *have been shown to be associated with increased PI3K activity and might contribute to cancer through inhibition of apoptosis [6,9]. Gene amplification/gain of gene copy is well accepted as a later event in tumor progression [18,19], as is somatic mutation [10]. To determine the relation between somatic mutation and gain of gene copy number of *PIK3CA *gene in breast cancer, we integrated our mutation and gene copy number data. As shown in Table 2, 19 (20.6%) of 92 cases had a *PIK3CA *gene mutation and 4 cases did not harbor a mutation but showed a gain of gene copy number. Overall, a quarter (23 of 92) of all breast tumors examined had either a mutation or gain of copy number of the *PIK3CA*. In addition, 15 of 19 mutations were identified in tumors without gain of copy number of *PIK3CA*, suggesting that somatic mutations are a major contributory factor in the *PIK3CA *signaling pathway. Only four cases in the whole study had both a mutation and gain of copy number of *PIK3CA*. We did not observe a significant association between somatic mutation and gain of *PIK3CA *gene copy number in 92 cases of breast tumors (Table 2). We suggest that further studies using larger number of cases be undertaken in order to determine whether somatic mutation and gene amplification are independent genetic alterations in breast cancer.

## Conclusion

The results from this study indicate that somatic mutation rather than gene amplification of *PIK3CA *is the main genetic alternation in breast cancer. The frequent and clustered mutations within *PIK3CA *make it an attractive molecular marker for early detection of breast cancer. In addition, the somatic mutations lead to activation of *PIK3CA *and also correlate with the activation of the PI3K/AKT pathway. Inhibition of *PIK3CA *can significantly induce apoptosis in cells with *PIK3CA *mutation. This suggests that *PIK3CA *might be a promising therapeutic target in breast cancer.

(During the writing of this manuscript, Bachman KE and colleagues published their results in *Cancer Biology and Therapy *[20]. They also reported more than 20% somatic mutations in breast cancer, a finding consistent with this study).

## Abbreviations

BAC = bacterial artificial chromosome; DAPI = 4',6-diamidino-2-phenylindole; FISH = fluorescence *in situ *hybridization; H & E = hematoxylin and eosin; PI3K = phosphatidylinositol 3-kinase.

## Authors' contributions

GW carried out PCR and sequencing reactions and prepared the manuscript. MX carried out primer design and PCR reactions. EM carried out real-time PCR reactions and prepared the manuscript. XH carried out FISH analysis. JW carried out apoptosis analysis. ZG carried out sequencing reactions. AC carried out sample preparation and real-time PCR. DG is the pathologist and carried out data analysis. SG coordinated FISH analysis. SS provided all breast cancer tissue and helped in designing the study. DS conceived of the study and participated in its design. BT coordinated all the studies. All authors read and approved the final manuscript.

## Figures and Tables

**Table 1:** Somatic mutation of *PIK3CA *in breast cancer cell lines and primary tumors

Exon	Nucleotide	Amino acid	Functional domain	Cell lines	Tumors	Total
9	C1616G	P539R	Helical	1 (BT20)	1	
9	G1624A	E542K	Helical		3	3
9	G1633A	E545K	Helical	2 (MDA361, MCF7)	8	10
9	A1634G	E545G	Helical		1	1
20	A3140G	H1047R	Kinase	2 (BT20, UACC893)	5	5
20	A3140T	H1047L	Kinase		1	
No. with mutations				5	19	22
No. samples screened				15	92	
Percent of cases with mutations				33.3	20.6	

**Table 2:** The relation of somatic mutation and gain of copy number of *PIK3CA *in breast cancer

	Mutation^-^	Mutation^+^	Total
Gain+	4	4	8
Gain-	69	15	84
Total	73	19	92

Fisher exact test, *P *= 0.054.
